# Sex differences in feeding behavior in rats: the relationship with neuronal activation in the hypothalamus

**DOI:** 10.3389/fnins.2015.00088

**Published:** 2015-03-30

**Authors:** Atsushi Fukushima, Hiroko Hagiwara, Hitomi Fujioka, Fukuko Kimura, Tatsuo Akema, Toshiya Funabashi

**Affiliations:** ^1^Department of Physiology, St. Marianna University School of MedicineKawasaki, Japan; ^2^Department of Physiology, Yokohama City University Graduate School of MedicineYokohama, Japan

**Keywords:** sex differences and hormone effects, feeding behavior, rats, CREB, melanin-concentrating hormone, orexin, hypothalamus

## Abstract

There is general agreement that the central nervous system in rodents differs between sexes due to the presence of gonadal steroid hormone during differentiation. Sex differences in feeding seem to occur among species, and responses to fasting (i.e., starvation), gonadal steroids (i.e., testosterone and estradiol), and diet (i.e., western-style diet) vary significantly between sexes. The hypothalamus is the center for controlling feeding behavior. We examined the activation of feeding-related peptides in neurons in the hypothalamus. Phosphorylation of cyclic AMP response element-binding protein (CREB) is a good marker for neural activation, as is the Fos antigen. Therefore, we predicted that sex differences in the activity of melanin-concentrating hormone (MCH) neurons would be associated with feeding behavior. We determined the response of MCH neurons to glucose in the lateral hypothalamic area (LHA) and our results suggested MCH neurons play an important role in sex differences in feeding behavior. In addition, fasting increased the number of orexin neurons harboring phosphorylated CREB in female rats (regardless of the estrous day), but not male rats. Glucose injection decreased the number of these neurons with phosphorylated CREB in fasted female rats. Finally, under normal spontaneous food intake, MCH neurons, but not orexin neurons, expressed phosphorylated CREB. These sex differences in response to fasting and glucose, as well as under normal conditions, suggest a vulnerability to metabolic challenges in females.

## Introduction

There is general agreement that the central nervous system in rodents differs between sexes due to the presence of gonadal steroid hormone during differentiation (Phoenix et al., 1959; Gorski and Barraclough, 1963). The organizing action of prenatally administered testosterone is evident on tissues that mediate mating behavior in female rodents (Arnold and Gorski, 1984). However, sexual differentiation of the brain is more complicated (McCarthy, 2008; Schwarz and McCarthy, 2008a; Nugent and McCarthy, 2011; Wu and Shah, 2011; Lenz et al., 2013) than once thought, even in rodents.

## Sexual differentiation of the hypothalamus: rodents and primates

For example, one apparent sexual difference of the hypothalamus is the mechanism for controlling gonadotropin secretion. Differentiation is certainly present in rodents (Butcher et al., 1974; Kalra and Kalra, 1983); however, in primates, the sexual differentiation of the pituitary function related to gonadotropin secretion is different from that in rodents (Karsch et al., 1973). Luteinizing hormone induction due to positive feedback from estrogen is evident in female, but not male, rodents (Kalra, 1993); although, in primates, both sexes secrete luteinizing hormone in response to estrogen (Karsch et al., 1973; Hodges, 1980). Estrogen positive feedback is capable of inducing luteinizing hormone secretion even in castrated human males, suggesting that exposure of the human brain to androgen during the early perinatal period does not completely induce a sexually dimorphic mechanism for controlling gonadotropin secretion (Barbarino and De Marinis, 1980). Alternatively, the apparent difference in sexual differentiation between primates and rodents may be due to differences between the hypothalamus- and pituitary-mediated control of gonadotropin secretion, since Fos is not expressed in response to gonadotropin-releasing hormone in monkeys (Witkin et al., 1994) but its expression is essential in rodents (Hoffman et al., 1990; Lee et al., 1990b,a).

## Sex differences in feeding behavior

**Figure 1:**
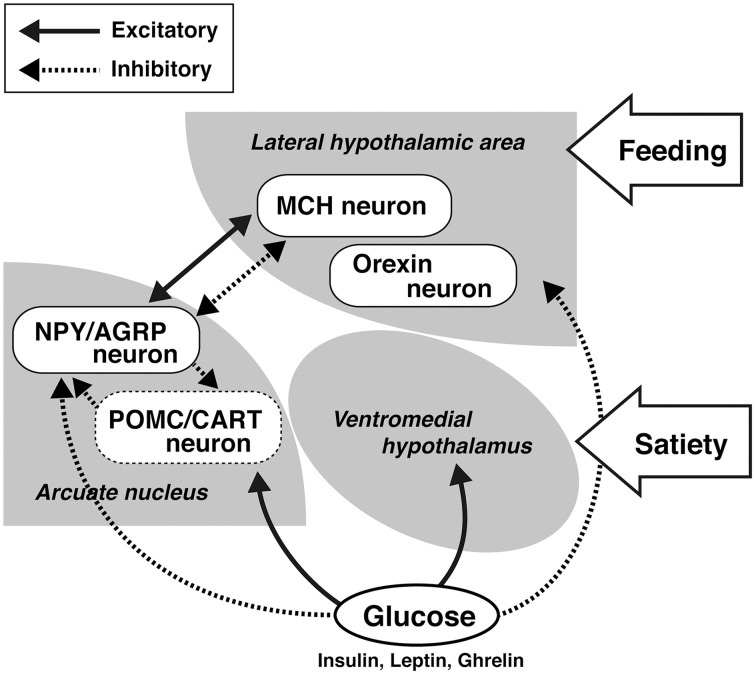
**Schematic of the control of feeding by glucose in the hypothalamus**.

On the other hand, there seems to be general sex differences in feeding among species. The hypothalamus is the center for controlling feeding behavior (Hervey, 1959; Bernardis and Bellinger, 1996). According to glucostatic theory, one of the factors controlling feeding is glucose (Mayer et al., 1952). As shown in Figure 1, glucose affects the control of feeding via a mechanism in the hypothalamus, which includes the ventromedial hypothalamus and the lateral hypothalamic area (LHA) (Oomura et al., 1964, 1974). Once it was determined that fat tissues secrete feeding inhibitory hormone in the response to energy consumption, the mechanism for feeding control drastically changed (Friedman, 2004). The hormone leptin is secreted from fat tissue and strongly inhibits feeding by controlling the neurons in the arcuate nucleus of the hypothalamus through its receptors (Friedman, 2009). Although the feeding control mechanism remains an important function of the hypothalamus (Anand and Brobeck, 1951; Hervey, 1959; Bernardis and Bellinger, 1996; King, 2005; Dietrich and Horvath, 2011), a recent hypothesis is that the first step involves the arcuate nucleus of the hypothalamus, which then controls the LHA and the periventricular nucleus (Koch and Horvath, 2014; Sousa-Ferreira et al., 2014).

There is a significant sex difference in taste preference (Valenstein et al., 1967). The effect of hypothalamic lesions on feeding also differs according to sex (Valenstein et al., 1969), suggesting there is a potential sex-specific feeding pattern in rats (Laviano et al., 1996). Metabolic states profoundly affect reproduction (Wade et al., 1996), and the responses to factors that alter feeding behavior, such as a high-fat diet (Uhley et al., 1997), fasting (Varma et al., 2001; Gayle et al., 2006), and leptin activity (Loh et al., 2011), are sex related. These sex-based differences in feeding behavior are probably the result of androgens present during sexual differentiation (Madrid et al., 1993; Schwarz and McCarthy, 2008b).

Importantly, these sex differences are also found in humans. In anorexia nervosa, there is a significant difference in morbidity between sexes (Geary, 2001; Schneider, 2006). The human hypothalamus is sexually differentiated (Swaab et al., 2001; Chung et al., 2002), as is food-related behavior in humans (Schneider, 2006; Zandian et al., 2011). Many behaviors in primates differ between sexes (Wilson and Davies, 2007; Hines, 2010) and may be related to the hormonal environment during sexual differentiation (Berenbaum and Beltz, 2011).

## Sex differences in feeding in rodents

**Figure 2:**
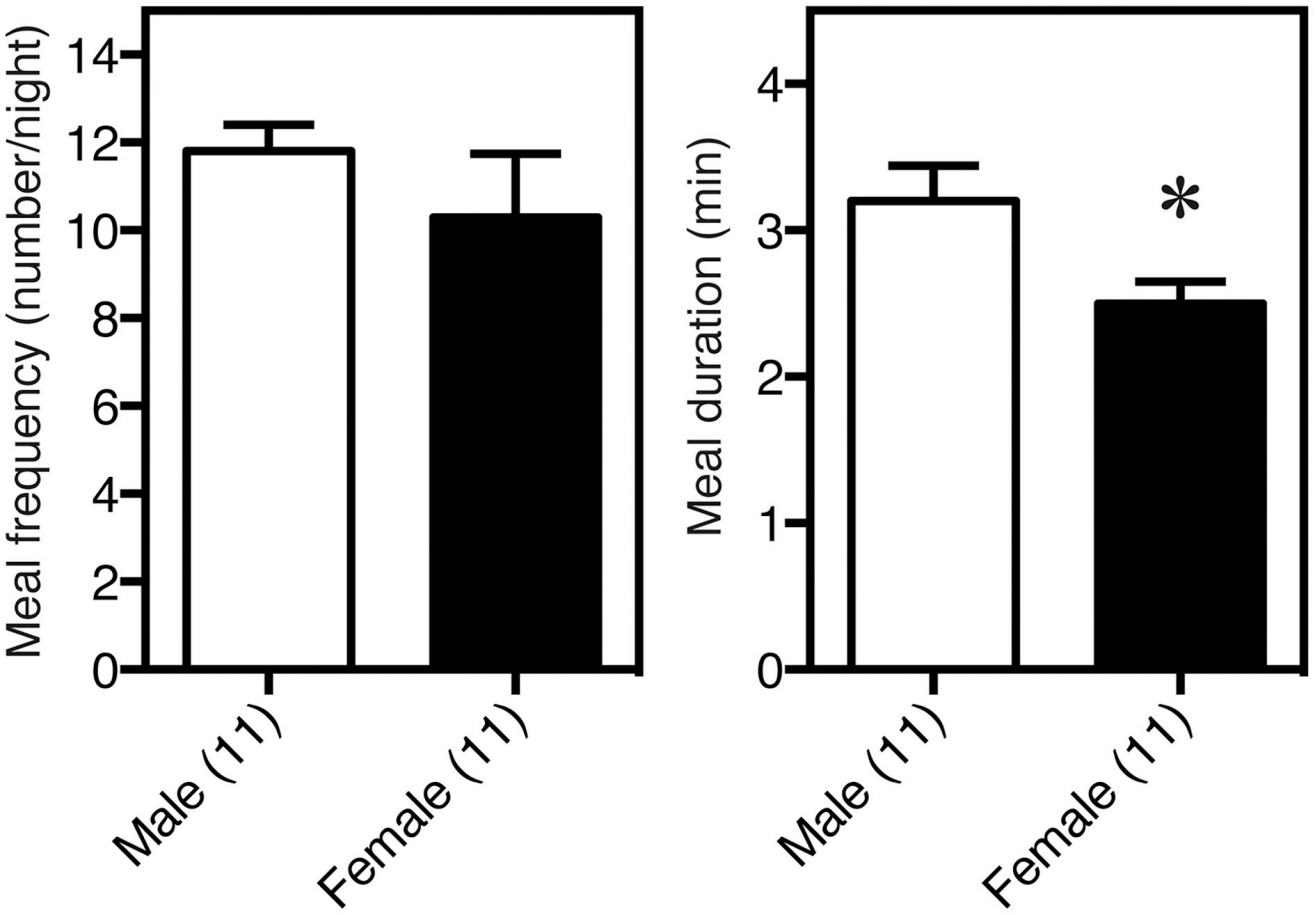
**Sex difference in feeding behavior, determined with an automated feeding pattern analyzer**. Meal duration, but not frequency, was significantly shorter in females than in males. ^*^P < 0.05.

**Figure 3:**
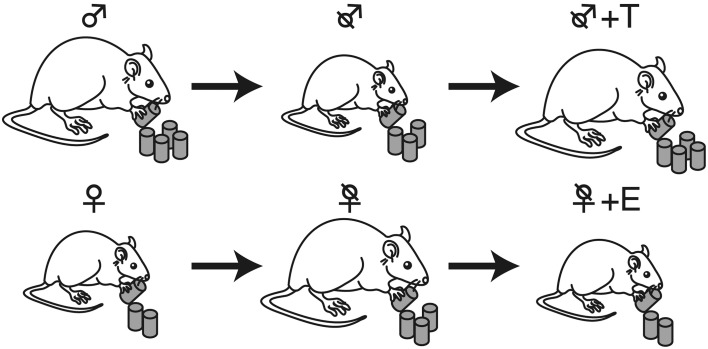
**Effects of gonadectomy and gonadal steroid hormones on feeding**. Males (♂) eat more than females (♀), but gonadectomy (\) had the opposite effect: castration of males resulted in weight loss because of decreased eating, while castration of females increased body weight due to hyperphagia. These changes were restored by testosterone (T) and estrogen (E) replacement, respectively.

The sex differences in the feeding behavior in rodents, including meal frequency and meal duration, were first determined using an automated feeding pattern analyzer (Meguid et al., 1990; Hyun et al., 1997). We confirmed that meal duration, but not meal frequency was significantly shorter in females than in males, as shown in Figure 2 (Funabashi et al., 2009) thus, there is a significant sex difference in feeding behavior. Male rodents are larger than females, in part due to the effects of testosterone (Petersen, 1978; Czaja, 1984; Asarian and Geary, 2006), as illustrated in Figure 3. On the other hand, estrogen reduces feeding (Eckel, 2004; Acosta-Martinez et al., 2007), probably via the ventromedial hypothalamus (Musatov et al., 2007; Butera, 2010; Xu et al., 2011) These effects of steroid hormones were demonstrated by gonadectomies (Kakolewski et al., 1968; Czaja, 1984). The body weight and food consumption in intact female rats were reduced when the effects of estrogen and progesterone were large (Tarttelin and Gorski, 1971). That is, at the time of ovulation when estrogen is high (Butcher et al., 1974), food intake was small and, as a result, body weight decreased in rats (Shimizu and Bray, 1993), bovine (Imakawa et al., 1986), and bamboo (Bielert and Busse, 1983) and rhesus monkeys (Kemnitz et al., 1989). These results illustrated that estrogen acts as a reducing factor of eating; therefore, estrogen is a target for reducing feeding behavior (Butera, 2010; Xu et al., 2011). Interestingly, male mice were more susceptible to high-fat induced obesity, known as experimentally induced obesity by diet (see review by Lai et al., 2014) than female mice (Nishikawa et al., 2007; Zammaretti et al., 2007; Hwang et al., 2010), and this was also the case with rats (Acosta-Martinez et al., 2007).

On the other hand, the effects of food deprivation in males and females are complicated. In general, adaption to food deprivation is important to survival in animals. Thus, rodents exhibit adaptive biochemical and physiological responses to food deprivation. For instance, rodents reduce metabolism when deprived of food (see review by Wang et al., 2006). Of course, the amount of food consumed after fasting, the rebound eating, is increased soon after. Although the total amount of food consumption remained higher than that seen in nonfasted rats, the rate of consumption declined for the next 9 h (Ji and Friedman, 1999). This means that, during starvation, energy expenditure is decreased and energy efficiency increased when refeeding occurs soon after fasting has stopped (Robin et al., 2008). Alternatively, rebound eating after caloric restriction is different among species (Evans et al., 2005).

Interesting evidence is that sex-specific fasting effects. Fasting for 12 h increased the total daily food consumption during the refeeding period in both male and female rats, but female rats show a greater increase in the first 24 h food intake than males. In addition, fasting induced a greater increase in plasma ghrelin levels in female rats compared with male rats (Gayle et al., 2006). Further, there were sex differences in the response to dietary disruption (Martin et al., 2007). We found that rebound eating after fasting was more prompt in female rats than in male rats (Funabashi et al., 2009).

## Phosphorylation of CREB in the hypothalamus

We sought to determine whether feeding-related peptides in neurons in the hypothalamus were activated. The Fos antigen (Sheng et al., 1990) and phosphorylation of cyclic AMP response element-binding protein (CREB) (Mayr and Montminy, 2001; Lonze and Ginty, 2002; Carlezon et al., 2005) are good markers for neural activation. Increasing cyclic AMP induced robust feeding (Gillard et al., 1998), suggesting that upregulation of a cyclic AMP-mediated cascade induces feeding. Indeed, neuropeptide Y acts as an orexinergic peptide, increasing CREB activity in the rat hypothalamus (Sheriff et al., 1997; Gillard et al., 1998) and downregulation of CREB induction attenuates leptin inhibition in neurons expressing neuropeptide Y (Shimizu-Albergine et al., 2001). Thus, CREB phosphorylation is a reliable marker for neuronal activity related to feeding behavior (Gayle et al., 2006; Martin et al., 2007; Funabashi et al., 2009). We attempted to attenuate CREB activity in the hypothalamus and evaluated the sex difference.

## Melanin-concentrating hormone and CREB phosphorylation in the LHA

Melanin-concentrating hormone (MCH) neurons in the LHA (Bittencourt et al., 1992) are involved in feeding behavior (Qu et al., 1996; De Lecea et al., 1998). Mice lacking MCH neurons are hypophagic (Shimada et al., 1998), and MCH receptor antagonists decrease feeding (Kowalski et al., 2004). Therefore, we predicted that sex differences in the activity of MCH neurons would be associated with feeding behavior. We determined the response to glucose of MCH neurons in the LHA using phosphorylated CREB as a marker of neural activity (Mogi et al., 2005). Intact male rats and female rats at various days of the estrous cycle were fasted for 48 h and injected with glucose. Thereafter, the rats' brains were analyzed by immunohistochemistry for MCH and phosphorylated CREB. Fasting for 48 h increased the percentage of MCH neurons in the LHA harboring phosphorylated CREB in both sexes, but glucose injection decreased the ratio of these double-stained cells more promptly in females than in males. Gonadectomy enhanced and attenuated the response of MCH neurons in males and females, respectively. Furthermore, steroid-hormone replacement in both males and females restored the response of MCH neurons to glucose. These results suggested that MCH neurons play an important role in sex differences in feeding behavior. It was later demonstrated that MCH stimulates feeding behavior and its receptor antagonist attenuates it in relation to palatability (Morens et al., 2005). Thus, MCH may be an important regulator of the intake of palatable foods such as sweet sugar water (Sakamaki et al., 2005; Baird et al., 2008; Fukushima et al., 2014), and MCH neurons are likely more active in females than in males. Estradiol may attenuate the feeding-stimulated effects of MCH in females (Messina et al., 2006), which vary during the estrous cycle (Santollo and Eckel, 2008).

## Orexin and CREB phosphorylation in the LHA

Since orexin neurons are also involved in feeding (Broberger et al., 1998; Sakurai et al., 1998; Bayer et al., 2005; Burdakov et al., 2005), we looked for a possible sex difference in the response of orexin neurons in the LHA to fasting (Funabashi et al., 2009). The experimental procedures were similar to those indicated above. Fasting increased the number of orexin neurons harboring phosphorylated CREB in female rats (regardless of the estrous day), but not in male rats; thus, there was a significant sex difference. Importantly, the action of orexin in feeding behavior is distinct from MCH. Glucose injection in fasted rats decreased the number of orexin neurons expressing phosphorylated CREB in female rats. These sex differences in the response of orexin neurons to fasting suggest a higher sensitivity of female hypothalamus to metablic cues. We also performed experiments under normal spontaneous food intake and found the MCH neurons, but not orexin neurons, expressed phosphorylated CREB. Again, attenuation seemed to occur faster in females than in males.

## Conclusions and future directions

**Figure 4:**
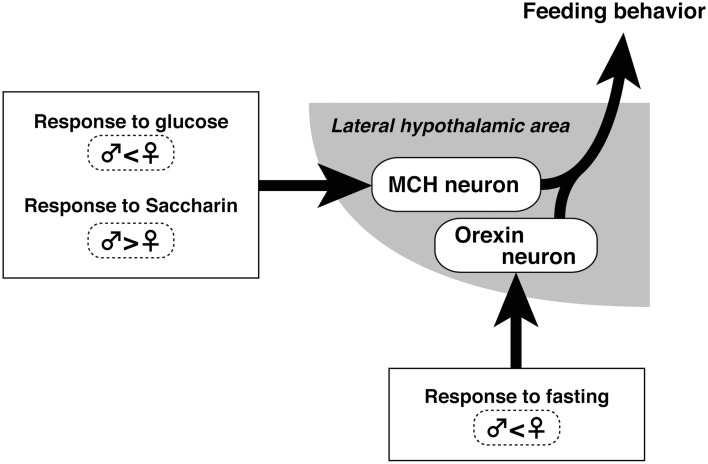
**Schematic of sex differences in MCH and orexin neuronal activity related to feeding control in the hypothalamus**.

We hypothesized that MCH neurons respond to nutrition-related feeding, but the feeding-related activity of orexin neurons is not evident unless hunger is accompanied by a bad emotion, such as that caused by fasting (Figure 4). Thus, the desire to eat under normal conditions does not drive orexin neurons, but it does drive MCH neurons. In line with this hypothesis, orexin inhibited pulsatile luteinizing hormone secretion under emotional conditions, but this effect was absent if food was available (Furuta et al., 2010). Future studies should determine what kind of emotion is associated with fasting and the neural basis for this mechanism.

### Conflict of interest statement

The authors declare that the research was conducted in the absence of any commercial or financial relationships that could be construed as a potential conflict of interest.
